# Development of an Affordable, Visual Feedback-Enhanced Auricular Field Block Model

**DOI:** 10.7759/cureus.88223

**Published:** 2025-07-18

**Authors:** Mel Ebeling

**Affiliations:** 1 Emergency Medicine, University of Cincinnati Medical Center, Cincinnati, USA

**Keywords:** auricle, medical education, model, nerve block, regional anesthesia, simulation

## Abstract

Injuries to the external ear commonly present to the emergency department and require early intervention to prevent serious complications, such as necrosis and ear deformities. Auricular field blocks are utilized to provide thorough anesthesia to the entire ear while preventing tissue swelling and anatomical distortion that is associated with the injection of local anesthetics directly into the external ear itself. Emergency medicine physicians must be competent and confident in their ability to perform auricular field blocks and manage lacerations to and hematomas of the external ear. This technical report details the development of an affordable, visual feedback-enhanced simulation model designed to educate on the procedural anatomy and technique of an auricular field block.

## Introduction

Auricular lacerations and hematomas are injuries to the external ear commonly encountered in the emergency department; indeed, the protruded nature of the pinna makes it vulnerable to injury. Due to the cartilaginous nature of the external ear, complications from these injuries can be severe, such as necrosis and long-lasting deformities (e.g., cauliflower ear), necessitating early management [1].

A peripheral nerve block of the external ear, commonly termed an "auricular field block," can be utilized in the repair of an auricular laceration or drainage of an auricular hematoma. An auricular field block anesthetizes the entire external ear by blocking the following four sensory nerves innervating the external ear: auriculotemporal nerve, greater auricular nerve, lesser occipital nerve, and auricular branch of the vagus nerve [2,3]. The superior portion of the pinna is innervated by the auriculotemporal nerve, the middle portion of the pinna is innervated by the lesser occipital nerve, and the lower portion of the pinna (including the lobule) is innervated by the greater auricular nerve. The auricular branch of the vagus nerve specifically provides sensory innervation to the concha and external auditory meatus. As with any sensory nerve innervation, there may be slight overlap or person-to-person variation, although these are the standard, anatomist-defined innervations. Utilizing an auricular field block for anesthesia of the external ear offers the advantage of eliminating the more severe pain and anatomical distortion/swelling that comes with direct injections of local anesthesia in the external ear itself. This is especially important for laceration repair, where careful alignment of the curved, sometimes tortuous structures of the pinna is necessary [4]. Moreover, injection of local anesthetic (e.g., lidocaine) with epinephrine directly into the external ear has been traditionally contraindicated due to the risk of tissue ischemia and necrosis, being that it is an area with end-arterial circulation.

Emergency medicine resident physicians are responsible for performing these careful laceration repairs and drainage of auricular hematomas. Competence in these procedures is an expectation reinforced by the 2022 Model of the Clinical Practice of Emergency Medicine, published by the American Board of Emergency Medicine [5]. Yet, there is no standardized curriculum by which emergency medicine resident physicians or medical students are educated on how to perform auricular field blocks; most likely, medical trainees are subjected to Halsted's "See One, Do One, Teach One" approach to procedural competency in the clinical environment, which appears to provide insufficient training while having implications for patient safety [6-8]. This technical report details the development of an affordable, visual feedback-enhanced simulation model designed to demonstrate the major innervations to the outer pinna and educate on the proper technique of an auricular field block. While generic injection simulators exist [9], there are no commercially available models designed for the auricular field block with the anatomy in mind. Moreover, simulation-based education can be exceedingly expensive, and the prerequisite possession of manufactured simulation equipment may still preclude some educators from being able to develop such a model [10,11]. Outside of the commercial realm, Eyre and Dobiesz previously developed a task-trainer for facial and dental regional anesthesia; however, it required the modification of an existing commercial simulation manikin task-trainer and was not equipped to educate on auricular blocks [12]. To the author's knowledge, the model detailed below is the first truly low-cost, visual feedback-enhanced model specifically designed for auricular field blocks.

## Technical report

Required materials and equipment

The materials, tools, and equipment necessary for the construction of this model are displayed in Table 1. Material costs are also included, which could vary slightly by the location of purchase or over time. 

**Table 1:** Materials and tools/equipment necessary for model construction EPS: expanded polystyrene; PPE: personal protective equipment

Required materials	Tools/equipment
EPS foam head with ears (this one measured 8.8 × 6.2 × 10.3 inches) - $8.99	Pen/marker
Polycrylic™ Crystal Clear Topcoat (Minwax®, Cleveland, OH, USA), clear gloss, 8 oz container - $12.98	Electric hot knife
Liquid latex, 16 oz bottle - $7.95	Flat paintbrush (2)
Three flexible LED filaments, DC3V, 130 mm long - $5.97	Soldering iron with rosin core solder
Roll of copper tape, 1 inch wide, 25 mm thick - $7.59	Hot glue gun with glue sticks
One 1.5V 2×AA battery holder with on/off switch - $1.40	Electrical tape
One 10 mL Luer lock syringe - $0.16	PPE, such as leather gloves, eye protection, latex gloves (if allergic to latex), and a respirator
600V AC, 22-gauge silicone tinned copper wire, stranded - $0.13	
One sheet of plywood (ours measured 14 × 18 × ½ inches) - $12.50	
Master's Touch acrylic paint, warm grey, 4.1 fl oz tube - $5.99	
Dressmaker pins - $2.67	

Methods

**Figure 1:**
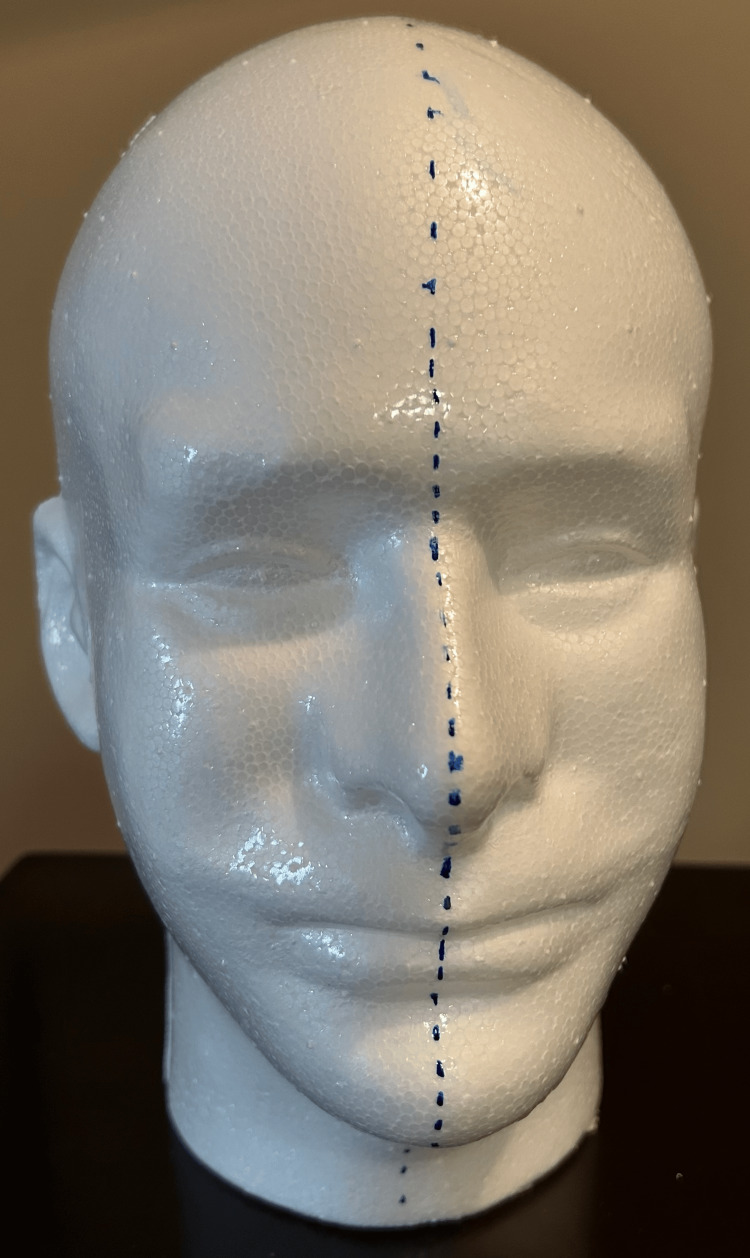
Midsagittal guideline on the foam head prior to cutting Polycrylic™-treated Styrofoam head with dotted line drawn by marker to aid in cutting the head in half. Note: both sides were treated with Polycrylic™.

Before beginning, ensure the use of personal protective equipment (PPE) and operate in a well-ventilated area. Using a paintbrush, apply a thin coat of Polycrylic™ (Minwax®, Cleveland, OH, USA) over the entirety of the foam head, except for the base, at zero, two, and four hours, for a total of three coats. After applying the third coat, allow the head to dry for 24 hours. This will prevent the liquid latex, applied later, from sticking to the Styrofoam. After the Polycrylic™ has dried, draw a midsagittal line along the length of the head, from the base of the neck anteriorly to the base of the neck posteriorly, in preparation for cutting the head in half (Figure 1).

**Figure 2:**
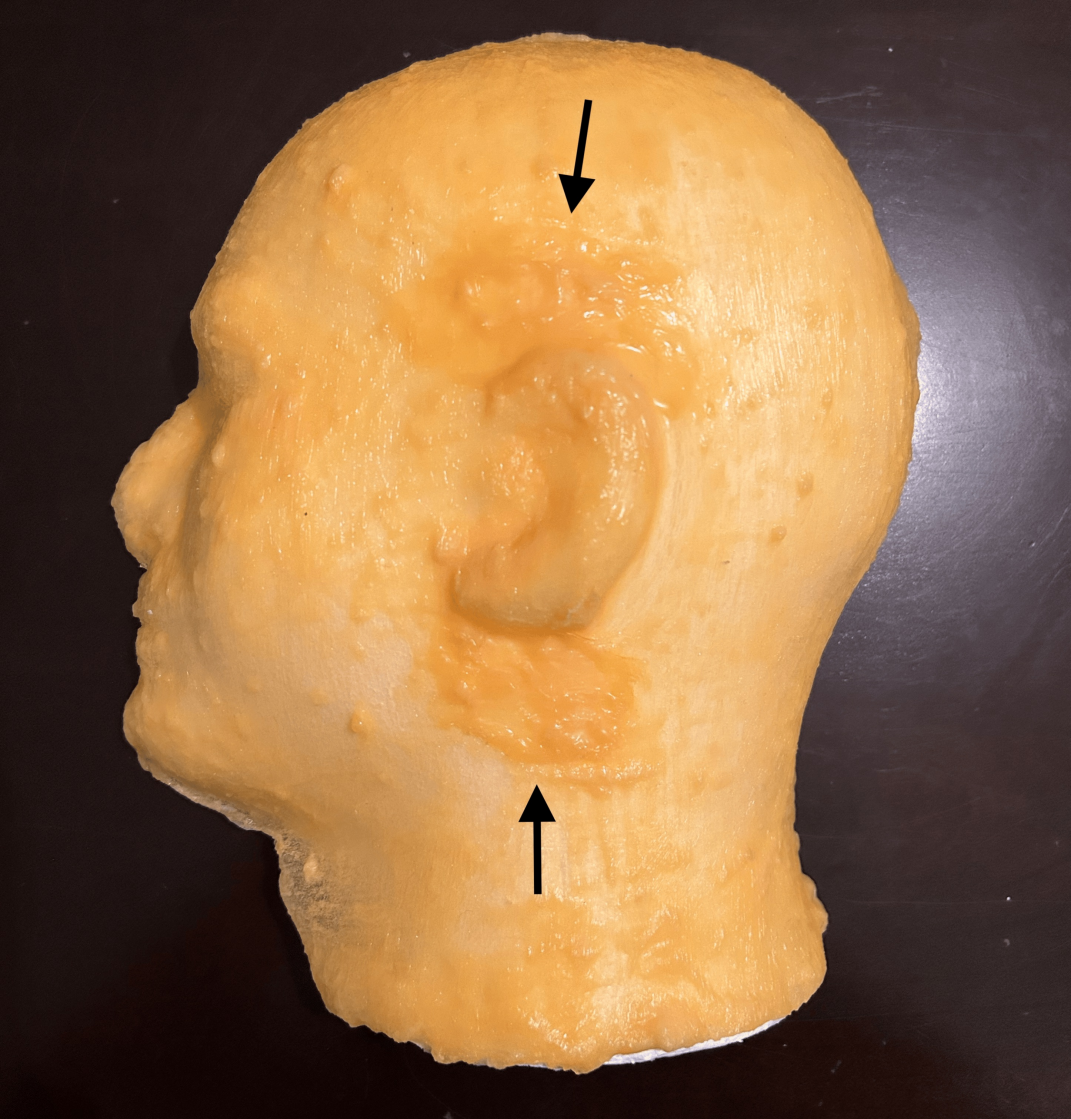
Completed latex skin Completed latex skin with arrows demonstrating the reinforced areas above and below the ear.

Cut the foam head in half along the midsagittal line using an electric hot knife. Only one half will be needed from here on out; the other half may be saved and used for an additional project or discarded. The left side of the head was used in this report. Position the foam head half, flat side down, on a stable surface. Using a flat paintbrush, apply a thin layer of liquid latex to the entire outer surface of the head, ensuring that all curves and crevices, particularly around the eye and ear, are appropriately coated. To create the latex "skin," apply a total of 14 coats of liquid latex, approximately one every hour to allow for adequate drying. Apply one to two additional thin coats of the liquid latex around the ear only for reinforcement, since this is the area that will receive repetitive needlesticks when used by learners (Figure 2).

**Figure 3:**
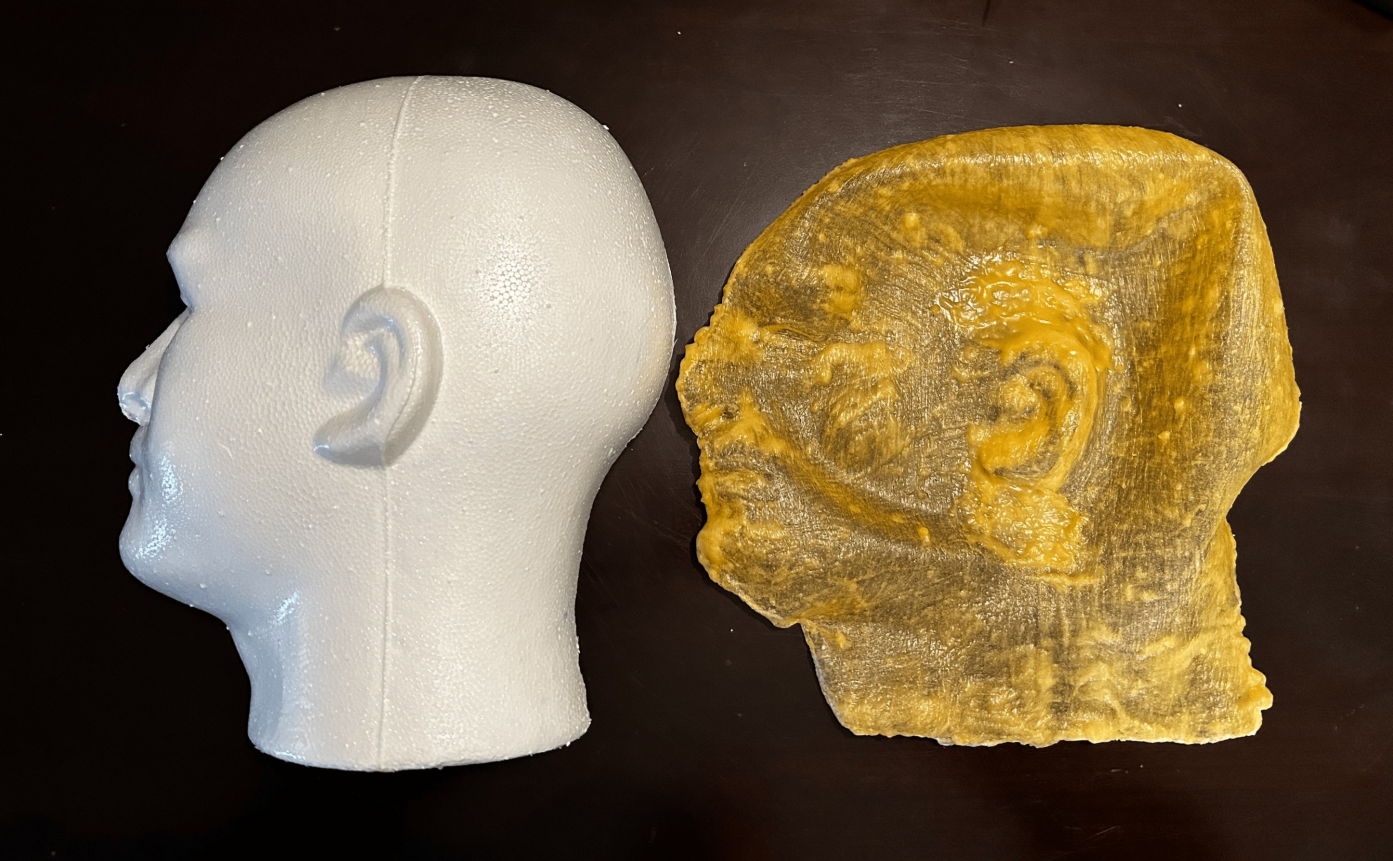
Integrity of the latex skin after removal from the foam head half Latex skin is flexible and translucent after removal from the foam head half.

Allow approximately one hour between coats. Once the liquid latex has fully dried, apply a single coat of the Master's Touch warm grey acrylic paint to its surface; this color was chosen for esthetic purposes to appear ethnic neutral. After the paint has dried (approximately one hour later), carefully remove the latex skin from the foam head half and set aside for later use (Figure 3).

**Figure 4:**
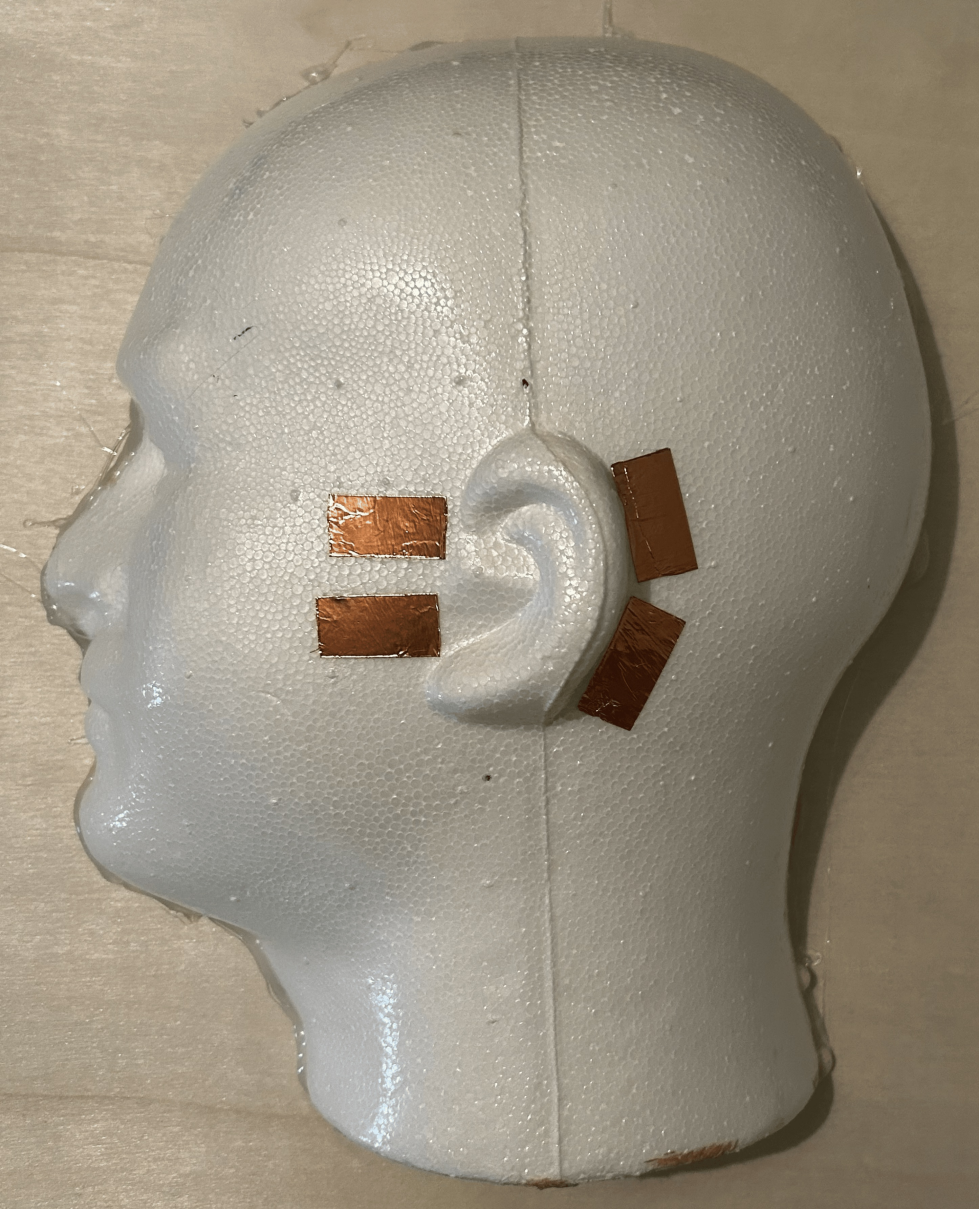
Placement of initial copper tape pieces Two pieces of conductive copper tape are oriented horizontally anterior to the ear, while two pieces of tape are oriented vertically immediately posterior to the ear.

Secure the foam head half to the middle of the plywood board using hot glue. Adhere four pieces of copper tape, each measuring 1 × 0.5 inch, to the foam head half, as shown in Figure 4.

**Figure 5:**
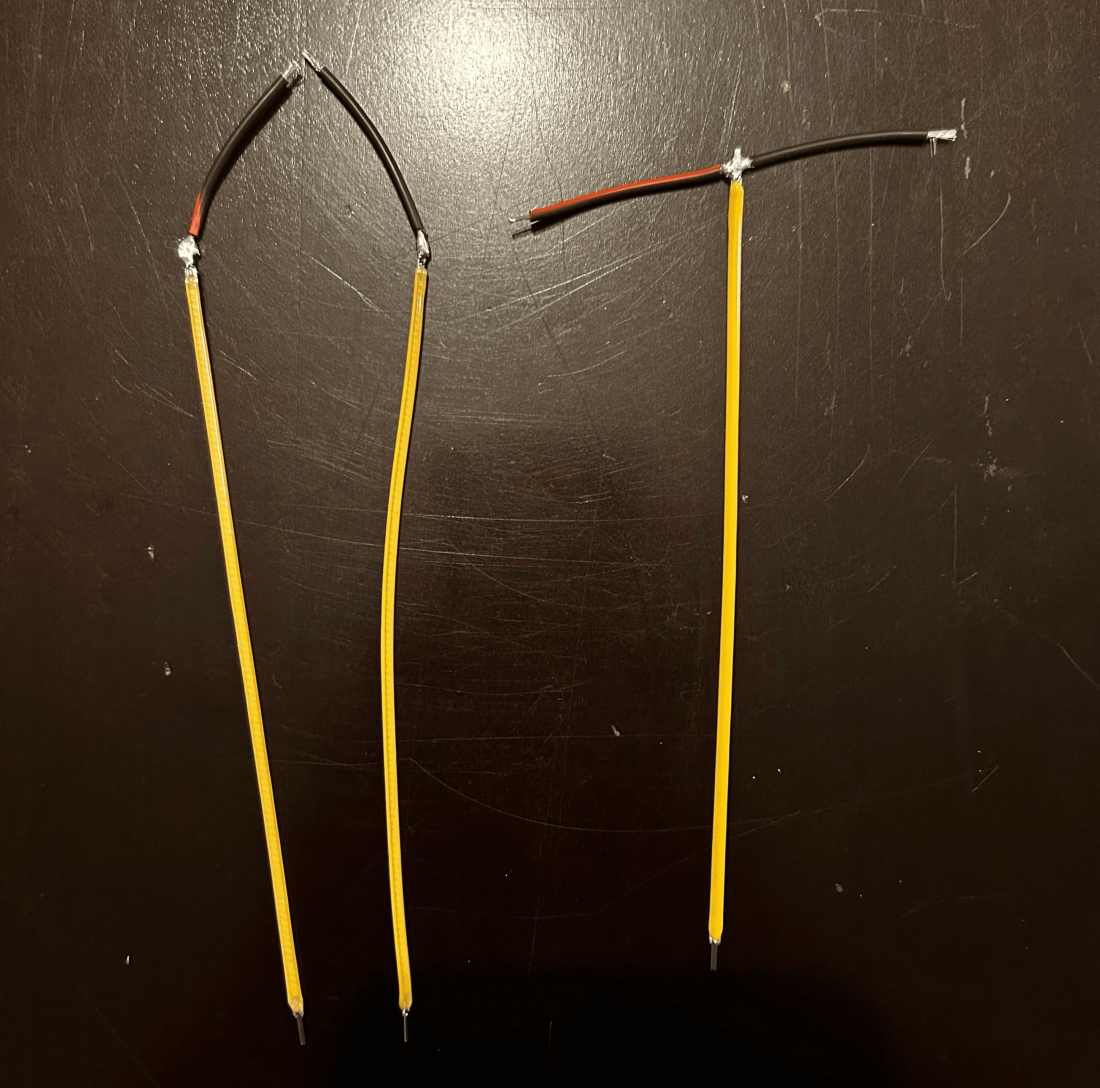
Wires soldered to LED filaments Two LED filaments are each soldered to a wire. The third LED filament is soldered to two wires to create a "T"-shape.

Cut four pieces of the 22-gauge wire, each measuring approximately 1.25 inches in length, and solder to the positive ends of the three LED filaments as shown in Figure 5.

**Figure 6:**
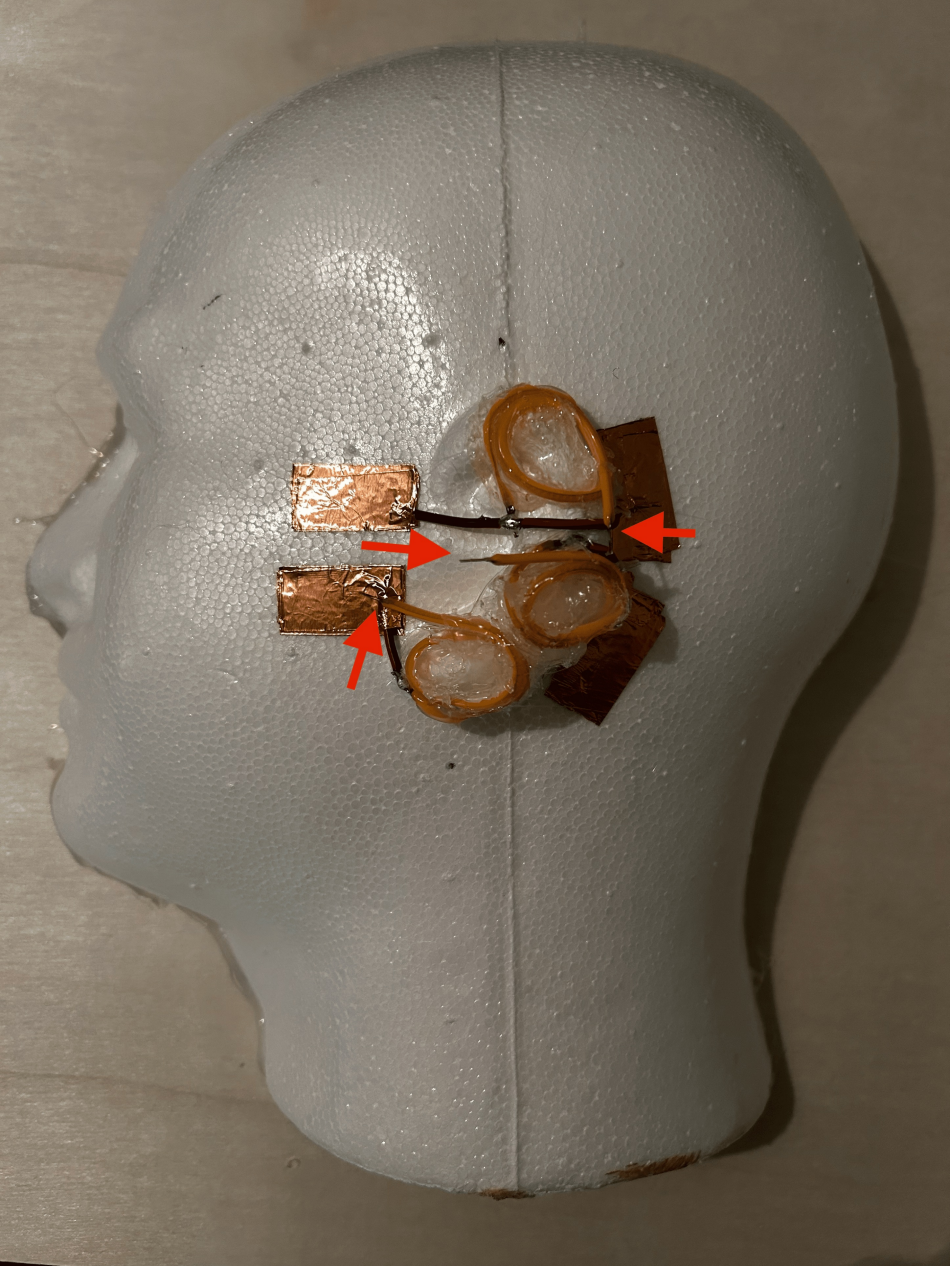
Location and orientation of the wire and LED filament placement on the foam head half Red arrows point to the location and orientation of the free negative ends of the three LED filaments.

Coil the three LED filaments upon themselves and hot glue them to the upper, middle, and lower portions of the ear on the foam head half, paying particular attention to the location and orientation of the conductive free ends of the copper wire and LED filament in relation to the preexisting copper tape. The two LED filaments each soldered to a single wire will be affixed to the middle and lower sections of the external ear and represent the innervations provided by the lesser occipital and greater auricular nerves, respectively. The third LED filament, the one soldered to two wires to form a "T"-shape, is intended for the uppermost part of the external ear. This LED filament will simulate the innervation of the auriculotemporal nerve when connected to the two superior pieces of copper tape, since the auriculotemporal nerve can be anesthetized by injecting both anterior and posterior to the ear, starting from the superior-most portion of the external ear. Using another four 1 × 0.5 inch pieces of copper tape, secure the conductive free ends of the four wires to the foam head half; place these new tape pieces over the existing ones, sandwiching the wires between the layers of tape. These steps are illustrated in Figure 6.

**Figure 7:**
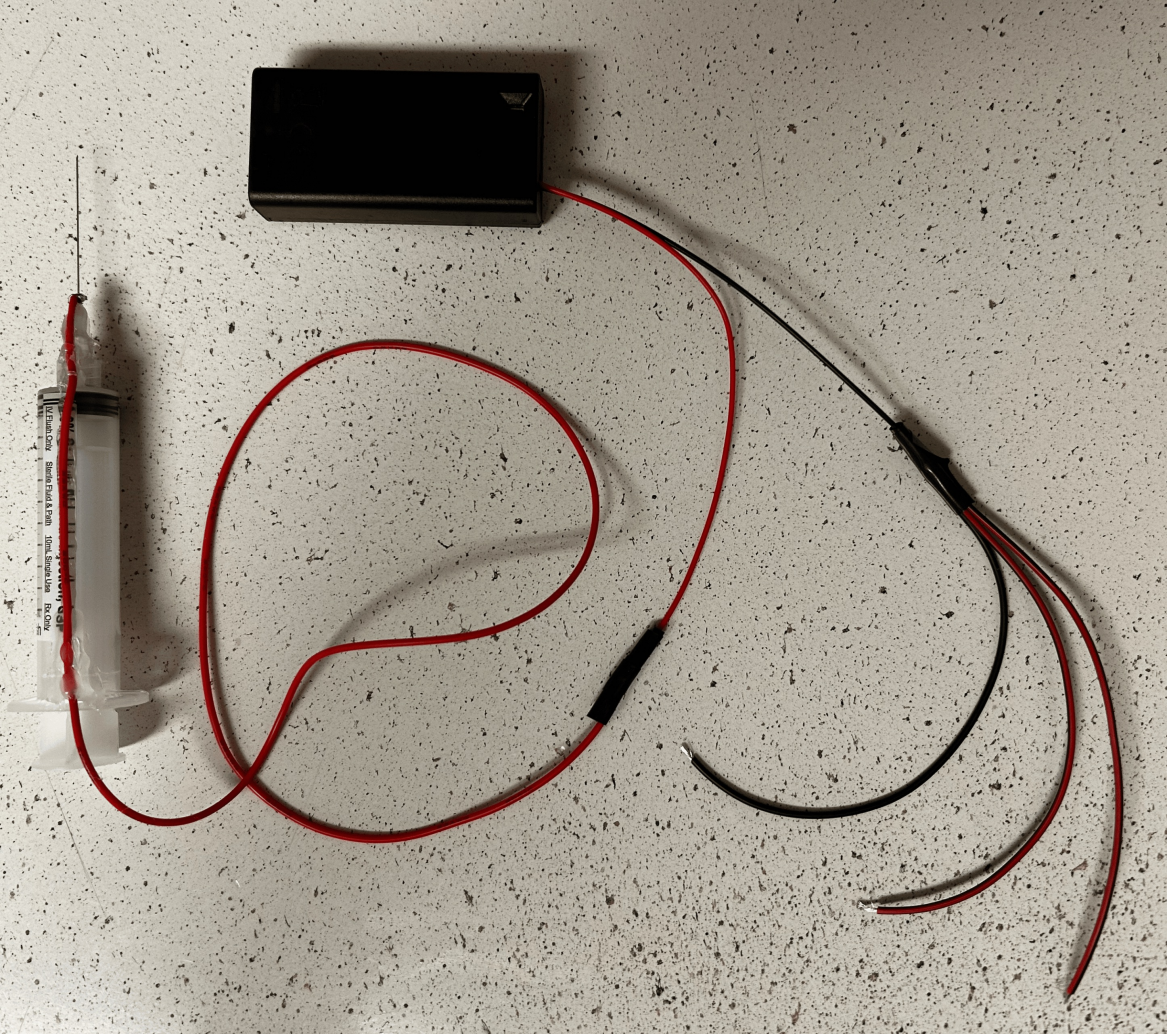
Partial circuit construction The needle is connected to the positive end of the battery holder through a wire. Three pieces of wire are shown connected to the negative end of the battery holder.

Screw the 27-gauge needle onto the Luer lock syringe. Next, cut a 24 inch piece of wire, and solder one end to the positive wire of the battery holder, securing this connection with electrical tape. Then, solder the other end of the wire to the base of the needle. Secure the wire running from the hub of the needle to the flange of the syringe to the barrel of the syringe using hot glue. Cut three pieces of wire, each measuring approximately 4 inches in length. Solder one end of each of the wires to the negative wire of the battery holder, securing this connection with electrical tape. Figure 7 displays the portion of the electrical circuit constructed.

**Figure 8:**
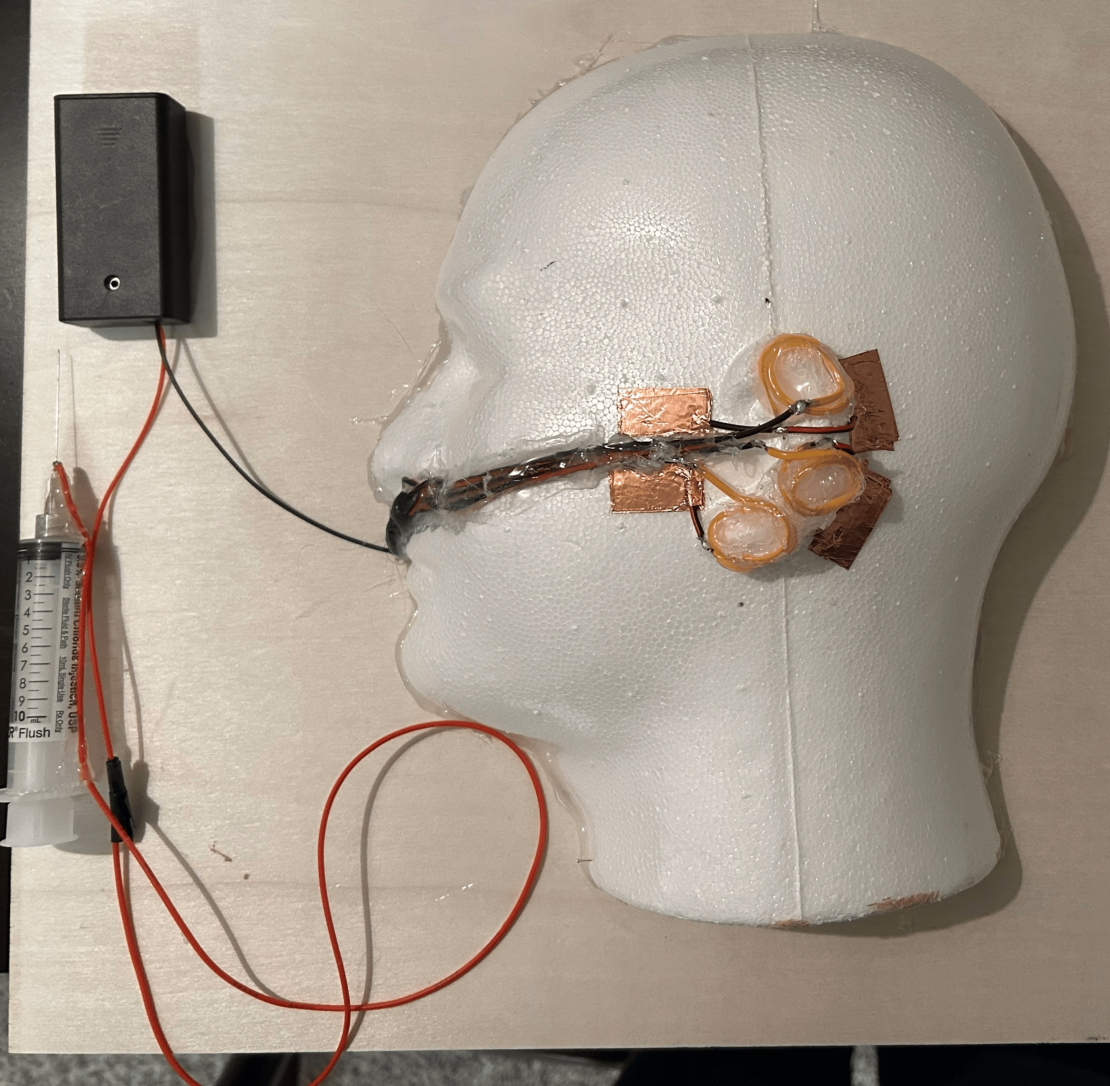
Fully constructed electrical circuit This electrical circuit is completed when the needle contacts any of the copper tape areas shown above.

Solder each of the three wires to the remaining free negative ends of the three LED filaments, ensuring a 1:1 connection (Figure 8).

**Figure 9:**
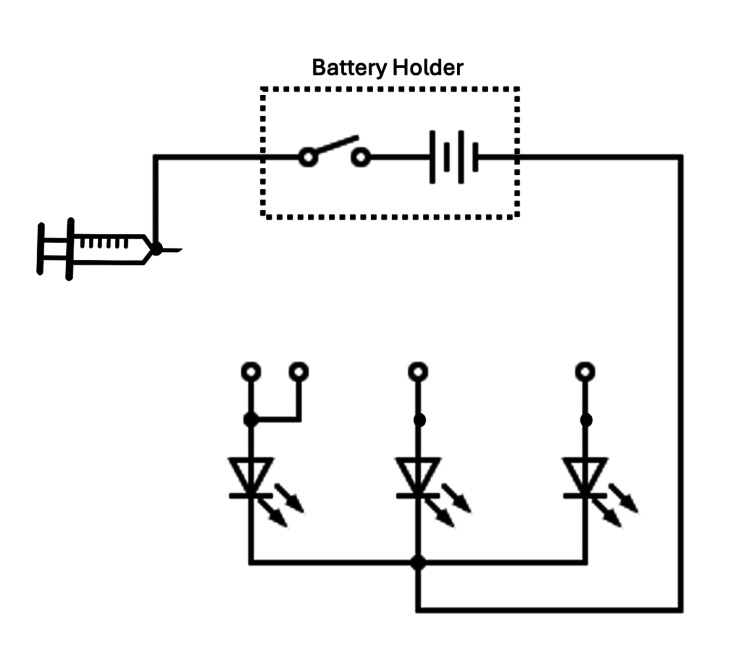
Circuit diagram This basic circuit diagram illustrates the essential connections required to complete the circuit.

Gently gather the three wires into a bundle and secure them to the foam head using hot glue. A basic circuit diagram for the model is shown in Figure 9.

Secure the battery holder face up to the upper left portion of the plywood board using hot glue. To expand the foam head half's conductive regions, overlap an additional ~1 × 0.5 inch piece of copper tape onto each of the four preexisting copper tape regions. Place the latex skin back over the foam head half and secure it to the head using dressmaker pins placed approximately every inch along the edge of the latex skin. For additional reinforcement around the ear, insert a couple of dressmaker pins along the posterior ridge of the ear, ensuring that they are pushed into the foam at a deep angle to avoid potentially puncturing the electrical components previously assembled.

**Figure 10:**
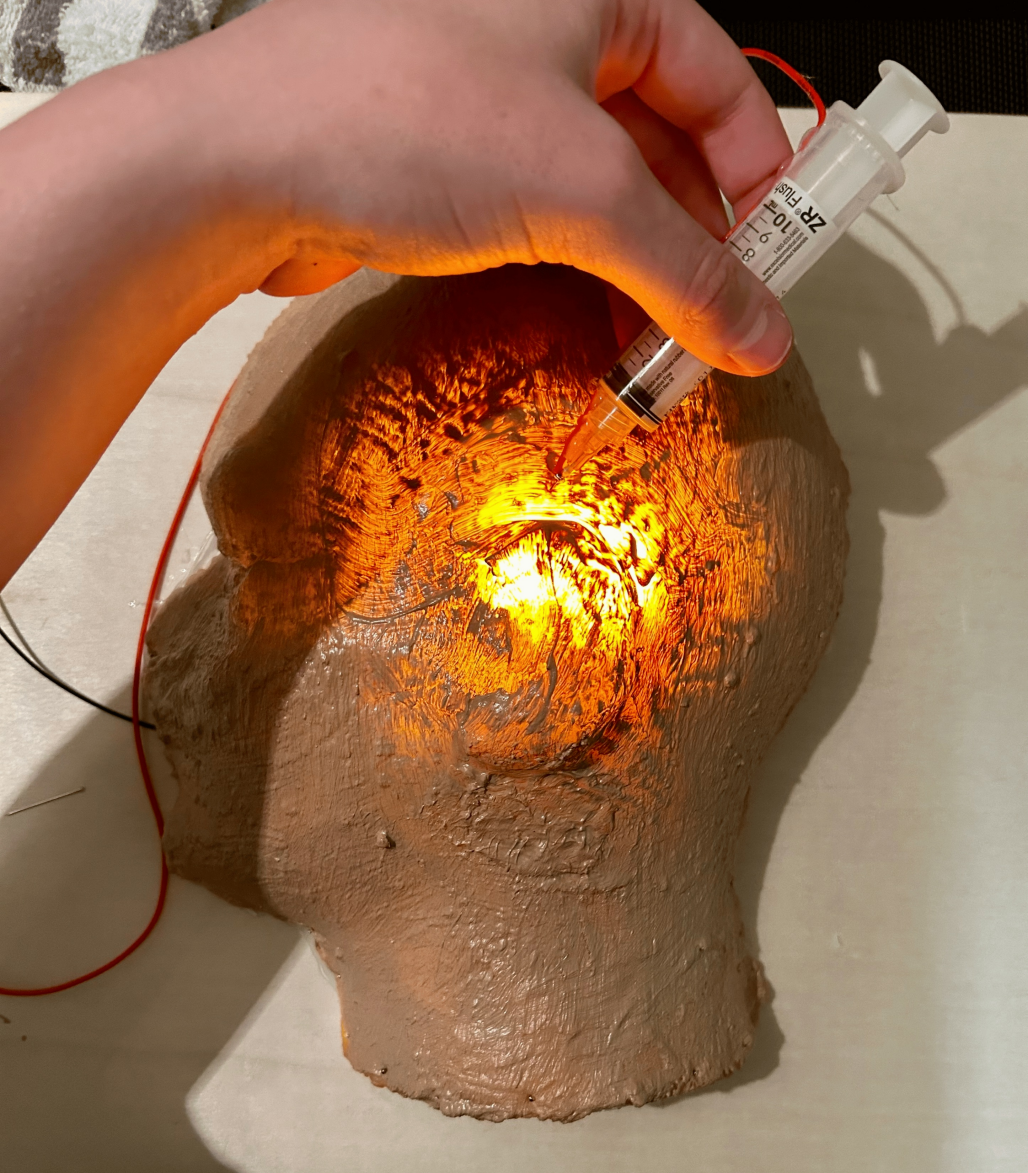
Model testing demonstrates block of sensation to the superior portion of the ear Light is concentrated around the portion of the external ear anesthetized by that part of the field block.

Insert two AA batteries in the battery holder and power it on. Then, guide the needle through the latex skin with the aim of contacting one of the areas with copper tape, which will complete the circuit. If done correctly, this action will result in light coming through the latex skin for as long as the needle is in contact with the copper tape and is meant to mimic success with auricular field blocking (Figure 10). Specifically, guiding the needle anteriorly from below the lobule towards the preauricular area simulates blocking the greater auricular nerve with local anesthetic. Guiding the needle posteriorly from the same starting point simulates blocking the lesser occipital nerve. Finally, guiding the needle both anteriorly and posteriorly from the area immediately above the superior-most portion of the pinna simulates blocking the auriculotemporal nerve.

## Discussion

This technical report describes the design and construction of a low-cost, visual feedback-enhanced auricular field block model for use by educators to improve the teaching of medical resident physicians and students on the procedure and anatomy involved in safe auricular field blocking. For physicians with preexisting knowledge of the procedure, this model could function solely for skills practice as well. The novelty of this model resides in its ability to provide anatomically specific visual feedback when simulating an auricular field block for less than $70. This compares favorably to generic commercial injection simulators, for example, that cost significantly more with no auricular landmarks, as previously stated [9]. Moreover, many of the materials came in sufficient quantities to be utilized in multiple additional constructions of this model if desired without the need for re-purchase. Despite its relative low fidelity, this model is durable; when stored in a sealed plastic container, the latex skin has maintained its integrity for over one and a half years.

The author acknowledges the presence of a few limitations of this model. Foremost, this model has not been extensively tested on cohorts of learners, limiting the ability to make evaluations on the real-world impact of this model. Second, some prior knowledge and experience with soldering is also necessary (or highly recommended), which may prevent some educators from developing a working model.

## Conclusions

Despite its limitations, this auricular field block model remains an affordable, accessible, and versatile educational tool for a variety of learner groups and could be used as just-in-time training for both resident physicians and medical students. Future directions for this project involve the robust evaluation of the model in increasing the confidence and competence of learners to optimally administer auricular anesthesia.
